# Novel Avian Influenza Virus (H5N1) Clade 2.3.4.4b Reassortants in Migratory Birds, China

**DOI:** 10.3201/eid2906.221723

**Published:** 2023-06

**Authors:** Jing Yang, Chunge Zhang, Yue Yuan, Ju Sun, Lu Lu, Honglei Sun, Heting Sun, Dong Chu, Siyuan Qin, Jianjun Chen, Chengbo Zhang, Xiyan Hao, Weifeng Shi, Wenjun Liu, George F. Gao, Paul Digard, Samantha Lycett, Yuhai Bi

**Affiliations:** Institute of Microbiology, Center for Influenza Research and Early-warning (CASCIRE), Chinese Academy of Sciences–The World Academy of Sciences Center of Excellence for Emerging Infectious Diseases, Chinese Academy of Sciences, Beijing, China (J. Yang, Chunge Zhang, J. Sun, W. Liu, G.F. Gao, Y. Bi);; University of Chinese Academy of Sciences, Beijing (J. Yang, Chunge Zhang, W. Liu, G.F. Gao, Y. Bi);; Shandong First Medical University, Taian, China (Y. Yuan, W. Shi, Y. Bi);; Shanxi Agricultural University, Taigu, China (J. Sun, Y. Bi);; University of Edinburgh, Edinburgh, UK (L. Lu, P. Digard, S. Lycett);; China Agricultural University, Beijing (Honglei Sun);; State Forestry and Grassland Administration, Shenyang, China (Heting Sun, D. Chu, S. Qin);; Wuhan Institute of Virology, Chinese Academy of Sciences, Wuhan, China (J. Chen);; Ordos Forestry and Grassland Development Center, Ordos, China (Chengbo Zhang);; Hohhot Center for Disease Control and Prevention, Hohhot, China (X. Hao)

**Keywords:** highly pathogenic avian influenza virus, H5N1, clade 2.3.4.4b, reassortant, genetic origin, phylogeography, antigenic variation, migratory bird, influenza, viruses, zoonoses, vaccine-preventable diseases, China

## Abstract

Two novel reassortant highly pathogenic avian influenza viruses (H5N1) clade 2.3.4.4b.2 were identified in dead migratory birds in China in November 2021. The viruses probably evolved among wild birds through different flyways connecting Europe and Asia. Their low antigenic reaction to vaccine antiserum indicates high risks to poultry and to public health.

Since the Gs/GD/96-lineage highly pathogenic avian influenza virus (HPAIV) (H5N1) was identified in 1996, H5 HPAIVs have evolved into divergent clades and caused continuous outbreaks in birds (*1*–*11*). Moreover, long-distance transmissions of H5 HPAIVs within a relatively short period indicate a crucial role of migratory birds in global spread of HPAIVs (*7*,*8*). Thus far, H5 viruses have undergone at least 4 waves of intercontinental transmission: H5N1 clade 2.2 during 2005–2006, H5N1 clade 2.3.2.1c during 2009–2010, H5N8 clade 2.3.4.4a and H5N1 clade 2.3.2.1c during 2014–2015, and H5Ny clade 2.3.4.4b during 2016–2017 (*2*–*8*).

Starting during 2020–2021, a new wave of HPAIV H5N1/H5N8 clade 2.3.4.4b outbreaks was reported in wild and domestic birds in Eurasia (*9*–*11*) and Africa (https://wahis.woah.org/#/event-management). Human cases of H5N1/H5N6/H5N8 infection were sporadically documented (https://www.who.int/teams/global-influenza-programme/avian-influenza/monthly-risk-assessment-summary), highlighting the zoonotic risk of H5 HPAIVs. Since 2021, H5 HPAIVs have caused at least 9 outbreaks in wild birds rather than poultry in mainland China (http://www.moa.gov.cn/gk/yjgl_1/yqfb; http://www.xmsyj.moa.gov.cn/yqfb). However, large outbreaks of H5N1 HPAIVs in domestic poultry were reported during 2021–2022 in Europe and the United States (https://www.cdc.gov/flu/avianflu/data-map-commercial.html) (A. Kandeil et al., unpub. data, https://doi.org/10.21203/rs.3.rs-2136604/v1). In this study, we explored the genetic origin, spread patterns, and antigenicity of H5N1 viruses identified from 2 dead migratory birds in China.

## The Study

We collected oral swab specimens and lung tissues from a dead whooper swan in northern China (Inner Mongolia) on November 3, 2021, and a deceased black swan in eastern China (Zhejiang) on November 15, 2021. We performed virus isolation in 10-day-old specific pathogen-free chicken embryos (*12*), then confirmed results by quantitative reverse transcription PCR (Mabsky Biotech, http://www.mabsky.com).

We isolated and Sanger sequenced 3 viruses, A/whooper swan/Northern China/11.03 IMEEDSAK1-O/2021 (Ws/NC/AK1-O/2021), A/whooper swan/Northern China/11.03 IMEEDSAK2-O/2021 (Ws/NC/AK2-O/2021), and A/black swan/Eastern China/11.15 ZJHZ74-Lg/2021 (Bs/EC/74-Lg/2021). We deposited whole genomes in NMDC (https://nmdc.cn; accession nos. NMDCN0000RD8–NMDCN0000RDV) and GISAID (https://www.gisaid.org; accession nos. EPI195500–EPI195523). We reconstructed phylogenetic trees for each gene of the 3 H5N1 isolates together with reference viruses from GISAID and the National Center for Biotechnology Information (https://www.ncbi.nlm.nih.gov/genomes/FLU/Database/nph-select.cgi), using the maximum-likelihood method with a general time-reversible model plus gamma distribution in RAxML 8.2.12 (https://cme.h-its.org/exelixis/web/software/raxml) (Appendix 1 Table). We reconstructed Bayesian time-resolved phylogenetic trees in BEAST 1.10.4 (https://beast.community/index.html) using the SRD06 model, the log-normal relaxed clock model, and the Skygrid coalescent model. We mapped spatial coordinates to the post burn-in time-scaled posterior trees using a Brownian motion continuous phylogeographic model. We mapped host type and hemagglutinin (HA) or neuraminidase (NA) subtype on each posterior tree by using a discrete trait phylogeographic model with BSSVS extension to infer the most likely ancestor with statistical support (Appendix 2).

We performed hemagglutination inhibitor (HI) assays (https://www.who.int/publications/i/item/manual-for-the-laboratory-diagnosis-and-virological-surveillance-of-influenza) to test the reactivities of antiserum of H5 Re-11/Re-13/Re-14 vaccines against these new H5N1 isolates and H5N8 HPAIVs identified in 2020 (*10*). Re-11 (A/duck/Guizhou/S4184/2017[H5N6], clade 2.3.4.4h) was used in poultry in China during December 2018–December 2021, whereas Re-13 (A/duck/Fujian/S1424/2020[H5N6], clade 2.3.4.4h) and Re-14 (A/whooper swan/Shanxi/4–1/2020[H5N8], clade 2.3.4.4b) have been deployed since January 2022 (http://www.moa.gov.cn/govpublic).

## Conclusions

**Figure 1:**
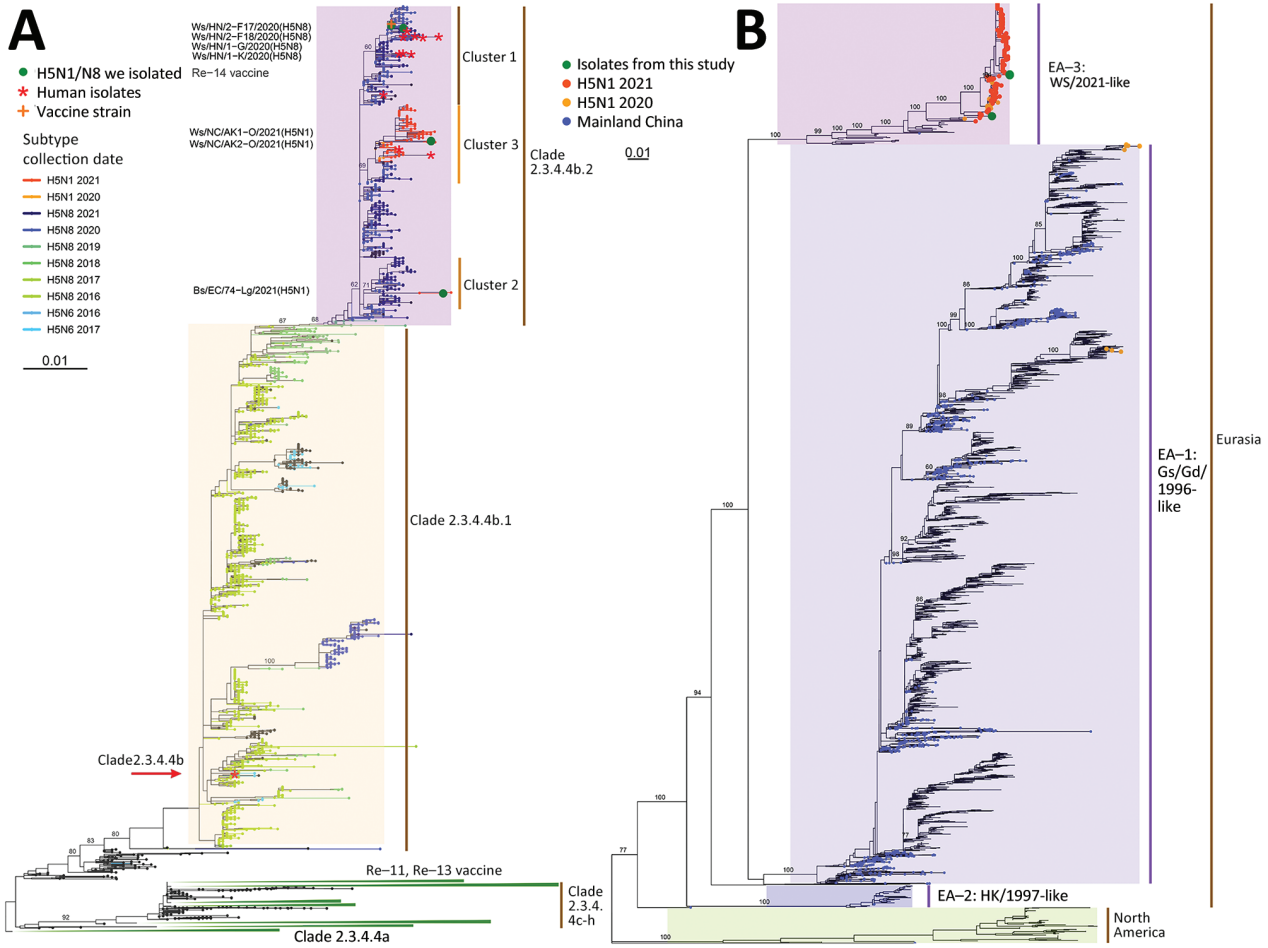
Phylogenetic trees for hemagglutinin genes of clade 2.3.4.4 H5Ny and neuraminidase genes of global H5N1 avian influenza viruses. A) Phylogeny of hemagglutinin genes of global clade 2.3.4.4 H5Ny avian influenza viruses. Solid green circles indicate H5N1 and H5N8 viruses from wild birds isolated in China; sequence names are listed next to corresponding circles. Red asterisks indicate human isolates. H5 vaccine seed strains used in mainland China are listed near the corresponding clades; orange cross indicates Re-14 vaccine. The major H5Ny subtypes within clade 2.3.4.4b are colored by their subtypes and collection dates. Clade 2.3.4.4b was divided into clade 2.3.4.4b.1 and clade 2.3.4.4b.2 because of >2.7% average pairwise nucleotide distance and >60% support for the 2 subclades. B) Phylogeny of global H5N1 neuraminidase genes. Colored circles indicate the novel H5N1 viruses from this study, H5N1 viruses from mainland China, and H5N1 viruses isolated in 2020 and 2021. Numbers on branches represent bootstrap support values for some major clades. Scale bar indicates number of nucleotide substitutions per site. Full phylogenetic trees of hemagglutinin genes of global H5Ny and neuraminidase genes of global H5N1 are provided at https://github.com/judyssister/globalH5N1_2021.

We obtained 3 H5N1 HPAIVs, Ws/NC/AK1-O/2021 and Ws/NC/AK2-O/2021 from a dead whooper swan in northern China and Bs/EC/74-Lg/2021 from a dead black swan in eastern China in November 2021. Consistent with the HPAIV signature of multiple basic amino acids on HA cleavage site, these H5N1 strains caused severe histopathologic changes in the wild birds (Appendix 2 Figure 1).

Phylogenetic analyses showed that all 3 H5N1 HA genes cluster in clade 2.3.4.4b.2 (Figure 1, panel A). Most H5 avian influenza viruses (AIVs) identified during 2020–2021 were in that clade, whereas H5N8 was the dominant subtype during 2019–2021, and H5N1 strains emerged in October 2020 and increased subsequently. In NA phylogeny, most H5N1 viruses identified during 2020–2021 including those 3 H5N1 viruses, were classified into the Eurasian lineage clade EA-3 (Figure 1, panel B). However, almost all H5N1 NA genes from mainland China were identified during 1996–2018 and are clade EA-1.

**Figure 2:**
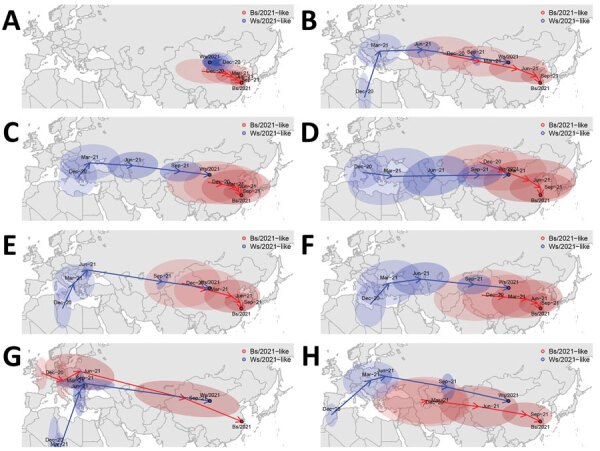
Spread patterns of all 8 gene segments of highly pathogenic avian influenza virus (H5N1), Bs/2021-like and Ws/2021-like reassortants, identified in migratory wild birds in China. Virus spread patterns reconstructed for 8 genes. A) polymerase basic 2 gene. B) polymerase basic 1 gene. C) polymerase acidic gene. D) hemagglutinin gene. E) nucleoprotein gene. F) neuraminidase gene. G) matrix gene. H) nonstructural protein gene. Blue indicates spread patterns of Ws/2021-like and red indicates spread patterns of Bs/2021-like H5N1. The spread patterns were adjusted by interpolating the ancestral space-time points by every 3 months from December 2020 through November 2021. Arrows represent the inferred ancestral locations at corresponding interpolated time (at 3-month intervals going back along their inferred transmission routes), and filled ellipses represent the 95% uncertainty of the inferred ancestral locations.

Given the HA phylogenetic relationships, we defined cluster 1, 2, and 3 in clade 2.3.4.4b.2 (Figure 1, panel A). Cluster 1 includes 4 H5N8 HPAIVs identified from wild birds in 2020 (*10*) and Re-14 vaccine strain. In cluster 2, Bs/EC/74-Lg/2021 was grouped with H5N1 viruses from Japan and South Korea, showing 99.3%–99.6% sequence identity. In cluster 3, Ws/NC/AK1-O/2021 and Ws/NC/AK2-O/2021 are identical (Appendix 2 Table 1) and clustered with H5N1 viruses from Europe, possessing 99.3% nucleotide identity. Most H5N1 viruses identified during 2020–2021 belong to cluster 3. Notably, 8 H5N6 and 1 H5N8 viruses that caused human infections (*13*) are found in cluster 1. Moreover, 2 human infections with cluster 3 H5N1 viruses were reported in the United Kingdom and United States during 2021–2022 (*14*). Therefore, this virus lineage poses a nonnegligible threat to public health, despite these viruses carrying non–mammalian-adapted molecular markers (Appendix 2 Table 2) and avian-type receptor-binding propensity (Appendix 2 Figure 2).

Current H5N1 viruses have resulted in substantial mortality in domestic and wild birds in Eurasia, Africa, and Americas (https://wahis.woah.org/#/event-management); however, they have only been identified in wild birds in mainland China. Compared with high HI antibody titers (256) between homologous antiserum and antigens of H5 vaccines, the recent H5N1/H5N8 viruses presented low HI titers (2–16) against Re-11/Re-13 antiserum (Table 1). In addition, cluster 1 H5N8 viruses had HI titers of 64 against Re-14 (cluster 1) antiserum, whereas HI titers for the H5N1 viruses were 128 for Bs/EC/74-Lg/2021 (cluster 2) and 32 for Ws/NC/AK1-O/2021 (cluster 3). This finding indicates lower antigenic identities between H5N1/H5N8 viruses circulating in wild birds and vaccines used in domestic poultry, even within the same clade. This antigenic variation may correlate to substitutions at antigenic sites (Table 2; Appendix 2 Tables 3,4, Figure 3).

**Table 1:** HI titers of H5N1 and H5N8 highly pathogenic avian influenza viruses from wild birds in China against antiserum of H5 Re-11, Re-13, and Re-14 vaccines*

Virus	HI titers of chicken antiserum against vaccine strains and H5N1/H5N8 isolates		
	Re-11, clade2.3.4.4h	Re-13, clade2.3.4.4h	Re-14, clade2.3.4.4b
Re-11, Dk/GZ/S4184/2017(H5N6)	256	256–512	128
Re-13, Dk/FJ/S1424/2020(H5N6)	32	256	2–4
Re-14, Ws/SX/4–1/2020(H5N8)	8	16	256
Bs/EC/74-Lg/2021(H5N1)	16	8	128
Ws/NC/AK2-O/2021(H5N1)	2–4	2–4	32
Ws/HN/1-K/2020(H5N8)	8	8	64
Ws/HN/1-G/2020(H5N8)	8	8	64

*H5 Re-11 vaccine was used in poultry in mainland China during December 2018–December 2021. H5 Re-13 and Re-14 vaccines have been deployed since January 2022. HI, hemagglutination inhibition.

**Table 2:** Amino acid substitutions on the hemagglutinin antigenic sites between H5N1 2021 and H5N8 2020 highly pathogenic avian influenza viruses and H5 vaccine seed viruses Re-11, Re-13, and Re-14 used in China*

Virus	Position of antigenic sites in hemagglutinin genes (H3 numbering)*															
	63	81	125	131	132	144	145	155	158	159	160	166	188	189	193	202
Re-11, clade2344h	D	R	R	T	S	V	A	T	N	D	A	M	A	E	N	V
Re-13, clade2344h	N	S	E	T	T	V	A	T	N	E	T	K	V	E	D	V
Re-14, clade2344b	D	R	S	E	T	A	P	I	N	D	A	I	A	E	N	I
Bs/EC/74-Lg/2021(H5N1)	D	R	N	E	T	A	P	I	N	D	A	I	A	K	D	I
Ws/NC/AK1-O/2021(H5N1)	D	R	S	E	T	A	P	I	D	D	A	I	A	K	N	I
Ws/NC/AK2-O/2021(H5N1)	D	R	S	E	T	A	P	I	D	D	A	I	A	K	N	I
Ws/HN/1-K/2020(H5N8)	D	R	S	E	T	A	P	I	N	D	A	I	A	E	N	I
Ws/HN/1-G/2020(H5N8)	D	R	S	E	T	A	P	I	N	D	A	I	A	E	N	I
Ws/HN/2-F17/2020(H5N8)	D	R	S	E	T	A	P	I	N	D	A	I	A	E	N	I
Ws/HN/2-F18/2020(H5N8)	D	R	S	E	T	A	P	I	N	D	A	I	A	E	N	I

*Positions of antigenic sites based on the H5 and H3 (sites A–E) antigenic sites.

Phylogenetic analyses uncovered that 3 novel H5N1 viruses could be classified into Ws/2021-like (Ws/NC/AK1-O/2021 and Ws/NC/AK2-O/2021) and Bs/2021-like (Bs/EC/74-Lg/2021) reassortants (Appendix 2 Figure 4). The viruses originated through separate reassortment events between H5N8 HPAIVs (obtaining HA and matrix [M] genes) and low pathogenic avian influenza virus pools (NA, polymerase basic 1, polymerase basic 2, polymerase acidic, nucleoprotein, and nonstructural protein genes) (Appendix 2 Figure 5–12). Phylogeographic analyses suggested that the H5N1 viruses spread to China by long-distance bird migration through various routes (Figure 2).

We reconstructed the genetic reassortment history for these H5N1 viruses (Appendix 2 Table 5, Figures 13, 14). For the Bs/2021-like reassortant, most gene segments group with viruses from wild Anseriformes in China or its adjacent areas, whereas the M gene likely originated from an HPAIV H5N8 ancestor from Eastern Europe in approximately May 2021 before import into China through bird migration. For the Ws/2021-like reassortant, 7 gene segments originated from Europe and were potentially transmitted by wild birds in February–August 2021, whereas a unique polymerase basic 2 gene originated from an early ancestry in 2017 with unknown origin but most closely related to a Russian H3N6 low pathogenic AIV. The ancestral states of most genes of the 2 reassortants indicate origins in wild Anseriformes and likely transmission through wild Anseriformes over the summer of 2021, whereas a few genes (e.g., Bs/2021-like M gene) potentially originated from domestic poultry. However, sampling bias in sequences might affect ancestral reconstruction by discrete trait phylogeographic models.

In conclusion, we identified 3 H5N1 HPAIVs in wild birds in autumn 2021, China. The antigenic divergence highlights the high-risk introduction of H5N1 circulating in wild birds to incompletely protected vaccinated flocks in China. The H5N1 viruses have experienced complicated reassortment during long-distance spread through various bird migration routes. Therefore, we call for international cooperation on AIV monitoring in migratory birds to help early identification and intervention of the emerging and reemerging AIVs with public health risks.

Appendix 1 GISAID sequences used in study of novel avian influenza virus (H5N1) clade 2.3.4.4b reassortants in migratory birds, China.

Appendix 2 Additional information for study of novel avian influenza virus (H5N1) clade 2.3.4.4b reassortants in migratory birds, China.
