# Supplementary material for: Novel Avian Influenza Virus (H5N1) Clade 2.3.4.4b Reassortants in Migratory Birds, China
Source: Emerg Infect Dis. 2023 Jun;29(6):1244–9. doi: 10.3201/eid2906.221723 (PMC10202878; doi:10.3201/eid2906.221723)
Supplement: Appendix 2 — Additional information for study of novel avian influenza virus (H5N1) clade 2.3.4.4b reassortants in migratory birds, China. [file 22-1723-Techapp-s2.pdf]

Article DOI: https://doi.org/10.3201/eid2906.221723

Novel Avian Influenza Virus (H5N1) Clade 2.3.4.4b Reassortants in Migratory Birds, China

Appendix 2

Methods

Phylogenetic analyses

To uncover the potential genetic origin and evolutionary position of the new H5N1 viruses, we constructed phylogenetic trees for each of the eight gene segments given their nucleotide sequences. In dataset 1, we collected all HA nucleotide sequences of H5Ny viruses and all NA sequences of H5N1 viruses from the GISAID EpiFlu database (*1*). In dataset 2, we also collected the first 500 most genetically related sequences to all eight gene segments of each of the three H5N1 viruses identified in this study from the Influenza Virus Resource database at the NCBI (https://www.ncbi.nlm.nih.gov/genomes/FLU/Database/nph-select.cgi) (*2*) and GISAID EpiFlu database by online BLAST tools. We combined these genetically similar sequences into a local database and ran a local BLAST against the local database to select the first 300 related sequences for the following analyses. We removed replicated sequences in each dataset according to DNA INSDC (international nucleotide sequence database collaboration) number and isolate names. We also removed sequences <75% full-length for each gene segment. We then aligned sequences of each gene segment with default parameters in MAFFT v7.490 (*3*). Maximum likelihood (ML) phylogenetic trees for each gene segment using the GTRGAMMA model with 1000 bootstraps in RAxML v8.2.12 were built (number of sequences 319–602 depending on segment, and 5789 sequences for NA gene of global H5N1), but for phylogeny

construction of global H5 (number of sequences is 13840), 100 bootstraps were used due to the computational complexity (*4*).

Phylogeographic analyses

We assessed the temporal signal of each gene segment in dataset 2 using the ML phylogenetic trees and collection dates of tree tips in Tempest v1.5.3 (http://tree.bio.ed.ac.uk/software/tempest/), resulting in datasets of 197–432 sequences spanning 2–20 years depending on the segment. The Bayesian time-resolved phylogenetic trees were reconstructed in BEAST 1.10.4 (*5*) using the SRD06 substitution model (an HKY substitution model and a site heterogeneity model using gamma distribution with four categories and two partitions for codon position 1+2 versus position 3) (*6*), an uncorrelated relaxed clock with a log-normal distribution, and a Skygrid coalescent model (*7*). A minimum of two independent Markov Chain Monte Carlo (MCMC) chains were run for each segment. Each chain consisted of 100,000,000 steps and was sampled every 10,000 steps, while the first 10% of samples were discarded as burn-in to achieve an effective sample size (ESS) >200. Later, a post burn-in subsample of 1,000 posterior trees for each segment was obtained for the location-annotated MCC tree visualization on the map. Spatial coordinates (latitudes and longitudes) were mapped to the time-scaled trees of each segment using a Brownian motion continuous phylogeographic diffusion model (*8*). In addition, host-type (containing domestic Anseriformes, domestic Galliformes, wild Anseriformes, other wild birds, mammals, and environment), HA subtype and NA subtype were also mapped on each tree using a discrete trait phylogeographic model with BSSVS extension to infer the most likely ancestor with statistical support (*9*). Phylogenetic trees were visualized and annotated in FigTree v1.4.4 (http://tree.bio.ed.ac.uk/software/figtree/). The MCC trees generated from the phylogeographic analyses are provided in a GitHub repository (https://github.com/judyssister/globalH5N1_2021).

Receptor binding assay

Receptor binding assays were performed using trisaccharide biotinylated glycan α-2,3-sialic acids (α2–3-SA) and α-2,6-sialic acids (α2–6-SA) receptors, respectively, according to previous studies (*10*). Two human strains, A/California/04/2009(H1N1) and A/Vietnam/1194/2004(H5N1), which have exclusive receptor binding preferences to α2–6-SA (human-type receptor) and α2–3-SA (avian-type receptor), respectively, were used as controls.

Histopathologic analysis of a dead black swan caused by H5N1 AIV

The lung, liver, and small intestine tissues of a freshly dead black swan infected by the H5N1 virus were fixed in 4% paraformaldehyde at room temperature. Serial coronal 4 μm thick sections were obtained and later stained with hematoxylin and eosin (H&E) by the Beijing One-Bio Technology Co., Ltd. The pathological changes were examined by light microscopy and scanned by a KF-PRO-120 Digital Pathology Scanner.

Homology modeling

To understand the positions of amino acid substitutions at antigenic sites, we inferred the structure of the ectodomain of the HA molecule of Ws/NC/AK1-O/2021 by homology modeling using the crystal structure of A/duck/Eastern China/L0321/2010 HA (H5N2; PDB ID: 7DEB) as a template in SWISS-model (*11*). The inferred 3-dimensional structure was annotated and visualized in PyMOL v2.5.2 (http://pymol.org/).

Results

Genetic characteristics of H5N1 isolates from wild birds

The molecular information at known functional sites can provide predictive power for certain phenotypic characteristics (e.g., pathogenicity, transmissivity, and risk of human infections) for novel AIVs (*12*). Typically, the polybasic amino acids at the HA cleavage site are considered as a genetic standard to identify H5 and H7 HPAIVs (*13*). In this study, the Bs/2021-

like H5N1 reassortant possessed PLRERRRKRGL, and the Ws/2021-like H5N1 reassortant and our H5N8 from wild birds in 2020 possessed PLREKRRKRGL at the cleavage sites, which indicated that these viruses belong to HPAIVs (Appendix 2 Table 2).

The combination of Q226L and G228S mutations in the H5 HA can increase the binding ability to the human-like sialic acid receptor (*14*), while a Q226L substitution in combination with N224K can also change the binding preference from avian-like to human-like receptors (*15*). The possession of 224N, 226Q, and 228G in the HA genes of our three H5N1 viruses suggested the viruses would exclusively bind to the avian-type receptor. In addition, as few as five amino acid substitutions (H110Y, T160A, Q226L, and G228S in the HA gene, and E627K in the PB2 gene) for A/Indonesia/5/2005(H5N1), and four substitutions (N158D, N224K, Q226L, and T318I in the HA gene) for A/Vietnam/1203/2004(H5N1) can confer airborne transmission for the H5 viruses among ferrets (*15*,*16*). The H5N1 reassortants we describe here have two mammalian-transmissible signatures in the HA gene, 158D and 160A. This indicates these viruses would most likely not transmit between ferrets.

Amino acid deletions in the NA stalk of AIVs can contribute to better adaption to terrestrial birds for viruses introduced by wild waterfowl and increase virulence in mammals (*17–19*). NA stalk deletions were not present in these H5N1 reassortants, which suggested they have not adapted to terrestrial birds thus far. Additionally, Q591K, E627K, and D701N mutations in the PB2 gene can confer better adaption for AIVs to mammals, such as boosting replication in mammalian cells and increasing pathogenicity in the mouse model (*20–24*). The 591Q, 627E, and 701D sequences in the PB2 gene of our H5N1 viruses indicated that they have not adapted to mammals. H274Y in the NA protein and S31N in M2 protein are NA- and M2-inhibitor resistance mutations, respectively (*25*,*26*). The 274H in NA and 31S in M2 genes of these H5N1 reassortants indicate they are sensitive to NA- and M2-inhibitors. Eight identical N-glycosylation sites are found in Re-14 and H5N1 viruses here, but one additional N-

glycosylation site is found in Bs/EC/74-Lg/2021, which could correlate to antigenic escape. Overall, we conclude that these novel viruses pose a low zoonotic/pandemic threat.

Necropsy and histopathological findings of a dead black swan caused by H5N1 virus

The infected black swan showed severe neurologic signs. At necropsy, serious lesions consisting of hemorrhages and necrosis in the lung, liver, and intestine were observed. Histologically, severe hemorrhage, edema, collapsed alveoli and inflammatory cell infiltration were found in the lung (Appendix 2 Figure 1). The intestine showed mucosal necrosis and hemorrhages. Focal necrosis was seen in the liver. Under high magnification, hepatocytes showed necrotic disintegration and inflammatory cells infiltrated around macrophages within the intervals among sinusoids.

The H5N1 isolates prefer avian-type receptors

The viral affinity to particular isoforms of the sialic acid receptors of host cells is one critical factor for host range of AIVs (*16*). The receptor binding assay showed all three H5N1 isolates have a receptor-binding preference to avian-type (α2–3-SA) receptors (Appendix 2 Figure 2). The avian-like receptor binding preference of the H5N1 is consistent with the molecular signatures observed at receptor binding sites, 224N, 226Q, and 228G (Appendix 2 Table 2).

References

1. Shu Y, McCauley J. GISAID: global initiative on sharing all influenza data—from vision to reality. Euro Surveill. 2017;22:30494. PubMed https://doi.org/10.2807/1560-7917.ES.2017.22.13.30494

2. Bao Y, Bolotov P, Dernovoy D, Kiryutin B, Zaslavsky L, Tatusova T, et al. The influenza virus resource at the National Center for Biotechnology Information. J Virol. 2008;82:596–601. PubMed https://doi.org/10.1128/JVI.02005-07

3. Katoh K, Standley DM. MAFFT multiple sequence alignment software version 7: improvements in performance and usability. Mol Biol Evol. 2013;30:772–80. PubMed https://doi.org/10.1093/molbev/mst010

4. Stamatakis A. RAxML version 8: a tool for phylogenetic analysis and post-analysis of large phylogenies. Bioinformatics. 2014;30:1312–3. PubMed https://doi.org/10.1093/bioinformatics/btu033

5. Suchard MA, Lemey P, Baele G, Ayres DL, Drummond AJ, Rambaut A. Bayesian phylogenetic and phylodynamic data integration using BEAST 1.10. Virus Evol. 2018;4:vey016. PubMed https://doi.org/10.1093/ve/vey016

6. Shapiro B, Rambaut A, Drummond AJ. Choosing appropriate substitution models for the phylogenetic analysis of protein-coding sequences. Mol Biol Evol. 2006;23:7–9. PubMed https://doi.org/10.1093/molbev/msj021

7. Hill V, Baele G. Bayesian estimation of past population dynamics in BEAST 1.10 using the Skygrid coalescent model. Mol Biol Evol. 2019;36:2620–8. PubMed https://doi.org/10.1093/molbev/msz172

8. Elliot MG, Mooers AØ. Inferring ancestral states without assuming neutrality or gradualism using a stable model of continuous character evolution. BMC Evol Biol. 2014;14:226. PubMed https://doi.org/10.1186/s12862-014-0226-8

9. Lemey P, Rambaut A, Drummond AJ, Suchard MA. Bayesian phylogeography finds its roots. PLOS Comput Biol. 2009;5:e1000520. PubMed https://doi.org/10.1371/journal.pcbi.1000520

10. Bi Y, Li J, Li S, Fu G, Jin T, Zhang C, et al. Dominant subtype switch in avian influenza viruses during 2016–2019 in China. Nat Commun. 2020;11:5909. PubMed https://doi.org/10.1038/s41467-020-19671-3

11. Waterhouse A, Bertoni M, Bienert S, Studer G, Tauriello G, Gumienny R, et al. SWISS-MODEL: homology modelling of protein structures and complexes. Nucleic Acids Res. 2018;46(W1):W296–303. PubMed https://doi.org/10.1093/nar/gky427

12. Bi Y, Zhang Z, Liu W, Yin Y, Hong J, Li X, et al. Highly pathogenic avian influenza A(H5N1) virus struck migratory birds in China in 2015. Sci Rep. 2015;5:12986. PubMed https://doi.org/10.1038/srep12986

13. World Organisation for Animal Health. High pathogenicity avian influenza (HPAI)-situation report 24. December 15, 2021 [cited 2022 Mar 1]. https://www.oie.int/en/document/high-pathogenicity-avian-influenza-hpai-situation-report-24

14. Stevens J, Blixt O, Tumpey TM, Taubenberger JK, Paulson JC, Wilson IA. Structure and receptor specificity of the hemagglutinin from an H5N1 influenza virus. Science. 2006;312:404–10. PubMed https://doi.org/10.1126/science.1124513

15. Imai M, Watanabe T, Hatta M, Das SC, Ozawa M, Shinya K, et al. Experimental adaptation of an influenza H5 HA confers respiratory droplet transmission to a reassortant H5 HA/H1N1 virus in ferrets. Nature. 2012;486:420–8. PubMed https://doi.org/10.1038/nature10831

16. Herfst S, Schrauwen EJ, Linster M, Chutinimitkul S, de Wit E, Munster VJ, et al. Airborne transmission of influenza A/H5N1 virus between ferrets. Science. 2012;336:1534–41. PubMed https://doi.org/10.1126/science.1213362

17. Matsuoka Y, Swayne DE, Thomas C, Rameix-Welti MA, Naffakh N, Warnes C, et al. Neuraminidase stalk length and additional glycosylation of the hemagglutinin influence the virulence of influenza H5N1 viruses for mice. J Virol. 2009;83:4704–8. PubMed https://doi.org/10.1128/JVI.01987-08

18. Zhou H, Yu Z, Hu Y, Tu J, Zou W, Peng Y, et al. The special neuraminidase stalk-motif responsible for increased virulence and pathogenesis of H5N1 influenza A virus. PLoS One. 2009;4:e6277. PubMed https://doi.org/10.1371/journal.pone.0006277

19. Cauldwell AV, Long JS, Moncorgé O, Barclay WS. Viral determinants of influenza A virus host range. J Gen Virol. 2014;95:1193–210. PubMed https://doi.org/10.1099/vir.0.062836-0

20. Subbarao K, Klimov A, Katz J, Regnery H, Lim W, Hall H, et al. Characterization of an avian influenza A (H5N1) virus isolated from a child with a fatal respiratory illness. Science. 1998;279:393–6. PubMed https://doi.org/10.1126/science.279.5349.393

21. Hatta M, Gao P, Halfmann P, Kawaoka Y. Molecular basis for high virulence of Hong Kong H5N1 influenza A viruses. Science. 2001;293:1840–2. PubMed https://doi.org/10.1126/science.1062882

22. Yamada S, Hatta M, Staker BL, Watanabe S, Imai M, Shinya K, et al. Biological and structural characterization of a host-adapting amino acid in influenza virus. PLoS Pathog. 2010;6:e1001034. PubMed https://doi.org/10.1371/journal.ppat.1001034

23. Zhou B, Pearce MB, Li Y, Wang J, Mason RJ, Tumpey TM, et al. Asparagine substitution at PB2 residue 701 enhances the replication, pathogenicity, and transmission of the 2009 pandemic H1N1 influenza A virus. PLoS One. 2013;8:e67616. PubMed https://doi.org/10.1371/journal.pone.0067616

24. Mok CK, Yen HL, Yu MY, Yuen KM, Sia SF, Chan MC, et al. Amino acid residues 253 and 591 of the PB2 protein of avian influenza virus A H9N2 contribute to mammalian pathogenesis. J Virol. 2011;85:9641–5. PubMed https://doi.org/10.1128/JVI.00702-11

25. Hay AJ, Wolstenholme AJ, Skehel JJ, Smith MH. The molecular basis of the specific anti-influenza action of amantadine. EMBO J. 1985;4:3021–4. PubMed https://doi.org/10.1002/j.1460-2075.1985.tb04038.x

26. Pinto LH, Holsinger LJ, Lamb RA. Influenza virus M2 protein has ion channel activity. Cell. 1992;69:517–28. PubMed https://doi.org/10.1016/0092-8674(92)90452-I

Appendix 2 Table 1. Percent identity of nucleotide sequences of H5N1 viruses identified in this study.

Viruses	HA	NA	PB2	PB1	PA	NS	MP	NP
A/whooper swan/Northern China/11.03 IMEEDSAK1-O/2021<br>A/black swan/Eastern China/11.15 ZJHZ74-Lg/2021	97.9	97.2	94.2	92.9	97.6	95.6	99.0	97.6

The A/whooper swan/Northern China/11.03 IMEEDSAK1-O/2021 and A/whooper swan/Northern China/11.03 IMEEDSAK2-O/2021 were identified from the same whooper swan. They have identical HA, NA, MP, NS, and PB2 genes but one different nucleotide site respectively in PA, NP, and PB1 genes.

Appendix 2 Table 2. Molecular characteristics on key functional sites for HA, PB2, NA, M2 proteins of H5N1 and H5N8 viruses.

	HA (H3 numbering)								PB2			NA (N2 numbering)				M2
Viruses	Cleavage site	110	158	160	224	226	228	318	591	627	701	247	252	274	Stalk deletion	31
A/black swan/Eastern China/11.15 ZJHZ74-Lg/2021(H5N1)	PLRERRRKR/GL	H	N	A	N	Q	G	T	Q	E	D	N	Y	H	No	S
A/whooper swan/Northern China/11.03 IMEEDSAK1-O/2021(H5N1)	PLREKRRKR/GL	H	D	A	N	Q	G	T	Q	E	D	N	Y	H	No	S
A/whooper swan/Northern China/11.03 IMEEDSAK2-O/2021(H5N1)	PLREKRRKR/GL	H	D	A	N	Q	G	T	Q	E	D	N	Y	H	No	S
A/whooper swan/Henan/CAS001-K/2020(H5N8)	PLREKRRKR/GL	H	N	A	N	Q	G	T	Q	E	D	N	Y	H	No	S
A/whooper swan/Henan/CAS001-G/2020(H5N8)	PLREKRRKR/GL	H	N	A	N	Q	G	T	Q	E	D	N	Y	H	No	S
A/whooper swan/Henan/CAS002-F17/2020(H5N8)	PLREKRRKR/GL	H	N	A	N	Q	G	T	Q	E	D	N	Y	H	No	S
A/whooper swan/Henan/CAS002-F18/2020(H5N8)	PLREKRRKR/GL	H	N	A	N	Q	G	T	Q	E	D	N	Y	H	No	S

Appendix 2 Table 3. Amino acid substitutions on the HA antigenic sites of H5N1/H5N8 HPAIVs and H5 Re-11, Re-13 and Re-14 vaccine seed viruses used in China given H5 antigenic sites.

	Position of antigenic sites in HA genes (H3 numbering)																						
Viruses	129	130	131	132	133	140	141	142	143	144	145	155	156	157	158	159	160	161	162	163	164	165	166
Re-11, clade2344h	N	H	**T**	**S**	S	P	Y	Q	G	**V**	**A**	**T**	K	K	**N**	**D**	**A**	Y	P	T	I	K	**M**
Re-13, clade2344h	N	H	**T**	**T**	S	P	Y	Q	G	**V**	**A**	**T**	K	K	**N**	**E**	**T**	Y	P	T	I	K	**K**
Re-14, clade2344b	N	H	**E**	**T**	S	P	Y	Q	G	**A**	**P**	**I**	K	K	**N**	**D**	**A**	Y	P	T	I	K	**I**
Bs/EC/74-Lg/2021(H5N1)	N	H	**E**	**T**	S	P	Y	Q	G	**A**	**P**	**I**	K	K	**N**	**D**	**A**	Y	P	T	I	K	**I**
Ws/NC/AK1-O/2021(H5N1)	N	H	**E**	**T**	S	P	Y	Q	G	**A**	**P**	**I**	K	K	**D**	**D**	**A**	Y	P	T	I	K	**I**
Ws/NC/AK2-O/2021(H5N1)	N	H	**E**	**T**	S	P	Y	Q	G	**A**	**P**	**I**	K	K	**D**	**D**	**A**	Y	P	T	I	K	**I**
Ws/HN/1-K/2020(H5N8)	N	H	**E**	**T**	S	P	Y	Q	G	**A**	**P**	**I**	K	K	**N**	**D**	**A**	Y	P	T	I	K	**I**
Ws/HN/1-G/2020(H5N8)	N	H	**E**	**T**	S	P	Y	Q	G	**A**	**P**	**I**	K	K	**N**	**D**	**A**	Y	P	T	I	K	**I**
Ws/HN/2-F17/2020(H5N8)	N	H	**E**	**T**	S	P	Y	Q	G	**A**	**P**	**I**	K	K	**N**	**D**	**A**	Y	P	T	I	K	**I**
Ws/HN/2-F18/2020(H5N8)	N	H	**E**	**T**	S	P	Y	Q	G	**A**	**P**	**I**	K	K	**N**	**D**	**A**	Y	P	T	I	K	**I**

The substitutions on H5 antigenic sites are shown in bold.

Appendix 2 Table 4. Amino acid substitutions on the HA antigenic sites of H5N1/H5N8 HPAIVs and H5 Re-11, Re-13 and Re-14 vaccine seed viruses used in China according to five antigenic sites A–E for H3 influenza virus.

Viruses	Position of antigenic sites in HA genes (H3 numbering)																						
	Site A																					Site B	
	122	123	124	125	126	127	129	132	133	134	135	136	137	138	140	141	142	143	144	145	146	155	156
Re-11, clade2344h	I	P	K	**R**	S	W	N	**S**	S	G	V	S	A	A	P	Y	Q	G	**V**	**A**	S	**T**	K
Re-13, clade2344h	I	P	K	**E**	S	W	N	**T**	S	G	V	S	A	A	P	Y	Q	G	**V**	**A**	S	**T**	K
Re-14, clade2344b	I	P	K	**S**	S	W	N	**T**	S	G	V	S	A	A	P	Y	Q	G	**A**	**P**	S	**I**	K
Bs/EC/74-Lg/2021(H5N1)	I	P	K	**N**	S	W	N	**T**	S	G	V	S	A	A	P	Y	Q	G	**A**	**P**	S	**I**	K
Ws/NC/AK1-O/2021(H5N1)	I	P	K	**S**	S	W	N	**T**	S	G	V	S	A	A	P	Y	Q	G	**A**	**P**	S	**I**	K
Ws/NC/AK2-O/2021(H5N1)	I	P	K	**S**	S	W	N	**T**	S	G	V	S	A	A	P	Y	Q	G	**A**	**P**	S	**I**	K
Ws/HN/1-K/2020(H5N8)	I	P	K	**S**	S	W	N	**T**	S	G	V	S	A	A	P	Y	Q	G	**A**	**P**	S	**I**	K
Ws/HN/1-G/2020(H5N8)	I	P	K	**S**	S	W	N	**T**	S	G	V	S	A	A	P	Y	Q	G	**A**	**P**	S	**I**	K
Ws/HN/2-F17/2020(H5N8)	I	P	K	**S**	S	W	N	**T**	S	G	V	S	A	A	P	Y	Q	G	**A**	**P**	S	**I**	K
Ws/HN/2-F18/2020(H5N8)	I	P	K	**S**	S	W	N	**T**	S	G	V	S	A	A	P	Y	Q	G	**A**	**P**	S	**I**	K

	Site B												Site C					Site D					
Viruses	157	158	159	160	186	188	189	190	193	194	196	197	50	53	54	275	278	174	201	202	203	204	205
Re-11, clade2344h	K	**N**	**D**	**A**	N	**A**	**E**	E	**N**	L	K	N	K	D	–	G	N	E	Y	**V**	S	V	G
Re-13, clade2344h	K	**N**	**E**	**T**	N	**V**	**E**	E	**D**	L	K	N	K	D	–	G	N	E	Y	**V**	S	V	G
Re-14, clade2344b	K	**N**	**D**	**A**	N	**A**	**E**	E	**N**	L	K	N	K	D	–	G	N	E	Y	**I**	S	V	G
Bs/EC/74-Lg/2021(H5N1)	K	**N**	**D**	**A**	N	**A**	**K**	E	**D**	L	K	N	K	D	–	G	N	E	Y	**I**	S	V	G
Ws/NC/AK1-O/2021(H5N1)	K	**D**	**D**	**A**	N	**A**	**K**	E	**N**	L	K	N	K	D	–	G	N	E	Y	**I**	S	V	G
Ws/NC/AK2-O/2021(H5N1)	K	**D**	**D**	**A**	N	**A**	**K**	E	**N**	L	K	N	K	D	–	G	N	E	Y	**I**	S	V	G
Ws/HN/1-K/2020(H5N8)	K	**N**	**D**	**A**	N	**A**	**E**	E	**N**	L	K	N	K	D	–	G	N	E	Y	**I**	S	V	G
Ws/HN/1-G/2020(H5N8)	K	**N**	**D**	**A**	N	**A**	**E**	E	**N**	L	K	N	K	D	–	G	N	E	Y	**I**	S	V	G
Ws/HN/2-F17/2020(H5N8)	K	**N**	**D**	**A**	N	**A**	**E**	E	**N**	L	K	N	K	D	–	G	N	E	Y	**I**	S	V	G
Ws/HN/2-F18/2020(H5N8)	K	**N**	**D**	**A**	N	**A**	**E**	E	**N**	L	K	N	K	D	–	G	N	E	Y	**I**	S	V	G

Viruses	Site D	Site E

Viruses	Position of antigenic sites in HA genes (H3 numbering)														
	206	207	208	217	218	219	220	62	63	78	79	80	81	82	83
Re-11, clade2344h	T	S	T	I	A	T	R	V	**D**	E	F	I	**R**	P	E
Re-13, clade2344h	T	S	T	I	A	T	R	V	**N**	E	F	I	**S**	P	E
Re-14, clade2344b	T	S	T	I	A	T	R	V	**D**	E	F	I	**R**	P	E
Bs/EC/74-Lg/2021(H5N1)	T	S	T	I	A	T	R	V	**D**	E	F	I	**R**	P	E
Ws/NC/AK1-O/2021(H5N1)	T	S	T	I	A	T	R	V	**D**	E	F	I	**R**	P	E
Ws/NC/AK2-O/2021(H5N1)	T	S	T	I	A	T	R	V	**D**	E	F	I	**R**	P	E
Ws/HN/1-K/2020(H5N8)	T	S	T	I	A	T	R	V	**D**	E	F	I	**R**	P	E
Ws/HN/1-G/2020(H5N8)	T	S	T	I	A	T	R	V	**D**	E	F	I	**R**	P	E
Ws/HN/2-F17/2020(H5N8)	T	S	T	I	A	T	R	V	**D**	E	F	I	**R**	P	E
Ws/HN/2-F18/2020(H5N8)	T	S	T	I	A	T	R	V	**D**	E	F	I	**R**	P	E

The substitutions on H3 antigenic sites are shown in bold.

Appendix 2 Table 5. Origin of the two novel Bs/2021-like and Ws/2021-like reassortants.*

		Ancestral	Time of origin						Ancestral	Host type probability¶			
Reassortants	Segment	type†	Median	95% HPD‡	HA	NA	Latitude	Longitude	host§	Dom Gal	Wild Ans	Wild other	Dom Ans
Bs/2021-like	PB2	MRCGA	Feb-21	(Oct-20, Jul-21)	H5	N1	35.039	115.893	Wild Ans	0.03	0.97	0	0
		MRCA	Apr-21	(Dec-20, Jul-21)	H5	N1	34.973	111.415	Wild Ans	0.03	0.97	0	0
	PB1	MRCGA	Jun-21	(Apr-21, Aug-21)	H5	N1	36.039	112.431	Wild Ans	0.07	0.93	0	0
		MRCA	Jul-21	(May-21, Sep-21)	H5	N1	35.267	116.604	Wild Ans	0.08	0.92	0	0
	PA	MRCGA	Jun-19	(May-18, Feb-20)	H5	N1	42.291	47.677	Dom Gal	0.66	0.27	0.03	0.03
		MRCA	May-21	(Jan-21, Aug-21)	H5	N1	34.823	115.92	Wild Ans	0.48	0.51	0	0.01
	HA	MRCGA	Jun-21	(Nov-21, Mar-22)	H5	N1	36.54	116.363	Wild Ans	0.39	0.58	0.02	0
		MRCA	Aug-21	(Dec-21, Apr-22)	H5	N1	35.29	119.93	Wild Ans	0.42	0.57	0.01	0
	NP	MRCGA	Jun-21	(Mar-21, Aug-21)	H5	N1	35.982	115.333	Wild Ans	0.28	0.71	0.01	0
		MRCA	Jul-21	(Apr-21, Sep-21)	H5	N1	35.226	118.38	Wild Ans	0.28	0.72	0	0
	NA	MRCGA	Apr-20	(May-19, Mar-21)	H5	N1	38.843	53.758	Wild Ans	0.24	0.47	0.28	0.01
		MRCA	Jul-21	(Mar-21, Aug-21)	H5	N1	35.313	116.577	Wild Ans	0.09	0.87	0.04	0
	MP	MRCGA	May-21	(Apr-21, Jul-21)	H5	N8	37.376	102.744	Dom Gal	0.63	0.35	0	0
		MRCA	Sep-21	(Jul-21, Oct-21)	H5	N1	35.137	112.058	Dom Gal	0.85	0.15	0	0
	NS	MRCGA	Feb-21	(Sep-20, Jun-21)	H5	N1	39.939	55.067	Wild Ans	0.2	0.8	0	0
		MRCA	Jul-21	(Apr-21, Oct-21)	H5	N1	35.261	107.565	Wild Ans	0.27	0.72	0	0
Ws/2021-like	PB2	MRCGA	Jul-17	(Oct-15, Feb-19)	H3	N6	39.802	126.235	Wild Ans	0	0.59	0	0.41
		MRCA	Oct-21	(Aug-21, Nov-21)	H5	N1	39.879	101.088	Wild Ans	0	1	0	0
	PB1	MRCGA	Apr-21	(Feb-21, Jun-21)	H5	N1	46.887	37.112	Wild Ans	0.23	0.77	0	0
		MRCA	Oct-21	(Sep-21, Nov-21)	H5	N1	40.347	96.064	Wild Ans	0	1	0	0
	PA	MRCGA	Mar-21	(Dec-20, May-21)	H5	N1	46.038	31.212	Wild Ans	0.07	0.92	0	0
		MRCA	Oct-21	(Aug-21, Nov-21)	H5	N1	40.294	95.796	Wild Ans	0	1	0	0
	HA	MRCGA	Mar-21	(Jul-21, Nov-21)	H5	N1	39.118	39.471	Dom Gal	0.66	0.33	0.01	0
		MRCA	Oct-21	(Jun-22, May-22)	H5	N1	39.904	94.895	Wild Ans	0	1	0	0
	NP	MRCGA	Jun-21	(Apr-21, Aug-21)	H5	N1	45.988	43.644	Wild Ans	0.03	0.97	0	0

Reassortants	Segment	Ancestral type†	Time of origin		HA	NA	Latitude	Longitude	Ancestral host§	Host type probability¶			
			Median	95% HPD‡						Dom Gal	Wild Ans	Wild other	Dom Ans
		MRCA	Oct-21	(Sep-21, Oct-21)	H5	N1	40.478	109.189	Wild Ans	0	1	0	0
	NA	MRCGA	Feb-21	(Sep-20, Apr-21)	H5	N1	44.859	35.22	Wild Ans	0.07	0.82	0.11	0
		MRCA	Oct-21	(Sep-21, Oct-21)	H5	N1	39.935	99.384	Wild Ans	0	1	0	0
	MP	MRCGA	Aug-21	(Aug-21, Sep-21)	H5	N1	45.415	39.516	Wild Ans	0.47	0.5	0	0.03
		MRCA	Oct-21	(Oct-21, Nov-21)	H5	N1	40.969	93.101	Wild Ans	0.02	0.98	0	0
	NS	MRCGA	May-21	(Mar-21, Jul-21)	H5	N1	49.937	40.477	Dom Gal	0.55	0.45	0	0
		MRCA	Oct-21	(Sep-21, Nov-21)	H5	N1	40.385	97.22	Wild Ans	0	1	0	0

*The estimated origin time, locations, host types and subtypes (HA and NA) of the grand most recent common ancestors (GMRCAs) and the most recent common ancestors (MRCAs) of two reassortants, Bs/2021-like from eastern China and Ws/2021-like from northern China.

†Two types of ancestors: the most recent common ancestor (MRCA) and the most recent common grand ancestor (MRCGA), which is the direct ancestor of the MRCA.

‡HPD is short for the highest posterior density interval.

§Four host types: Domestic Galliformes (Dom Gal), Wild Anseriformes (Wild Ans), Domestic Anseriformes (Dom Ans) and other wild bird types (Wild other).

¶The posterior probability of four ancestor host types at the corresponding ancestral nodes.

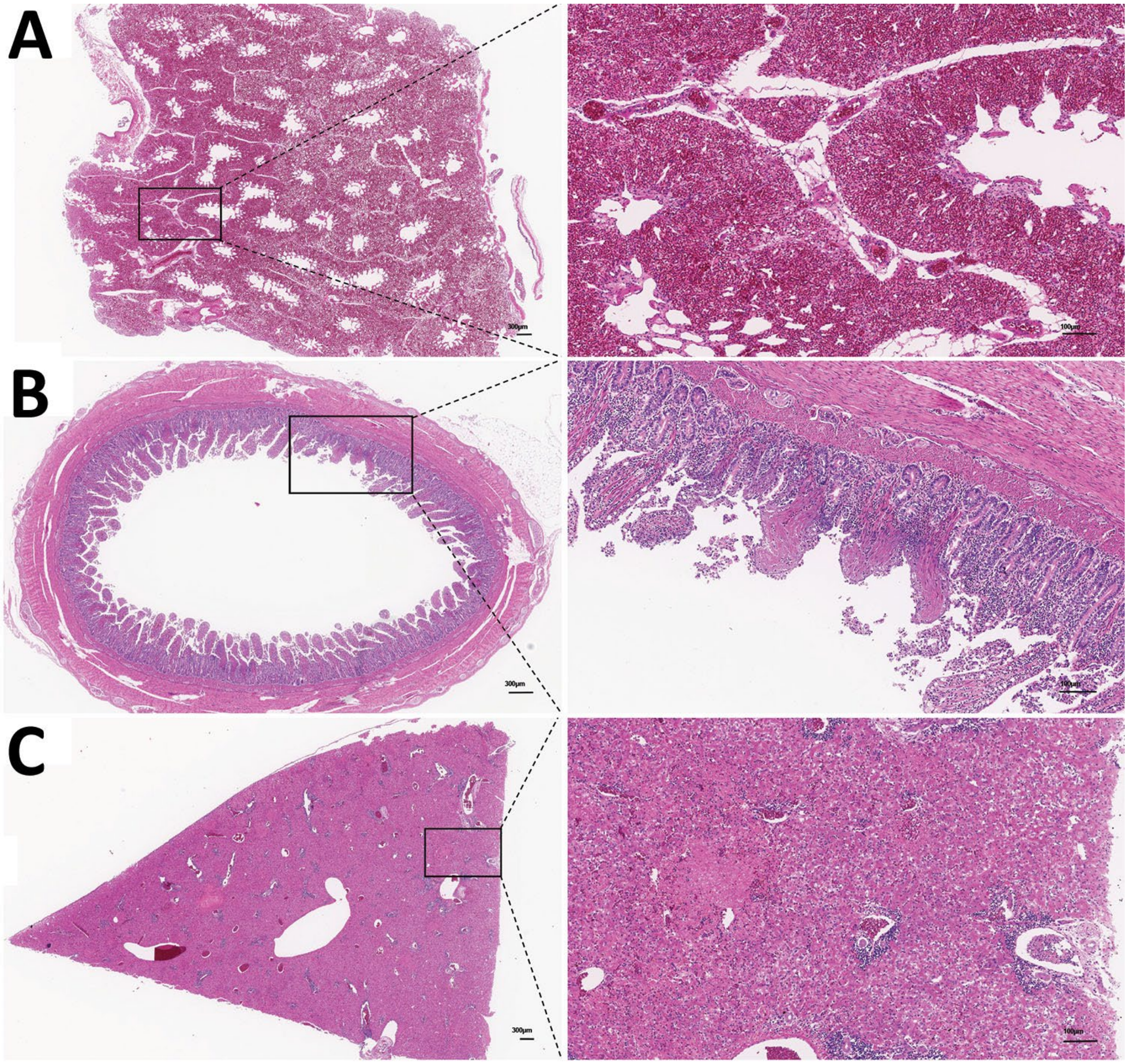

Appendix 2 Figure 1. Histopathologic changes in the tissues of a dead black swan infected by H5N1 virus. Histopathologic changes of (a) hemorrhages in the lung, (b) necrosis in the intestine, and (c) necrosis in the liver. The left panel of each sub-figure shows the panoramic image of corresponding tissues. The right panel shows an enlarged view with obviously histopathologic changes. Scale bars represent 300µm (left panel) and 100µm (right panel), respectively.

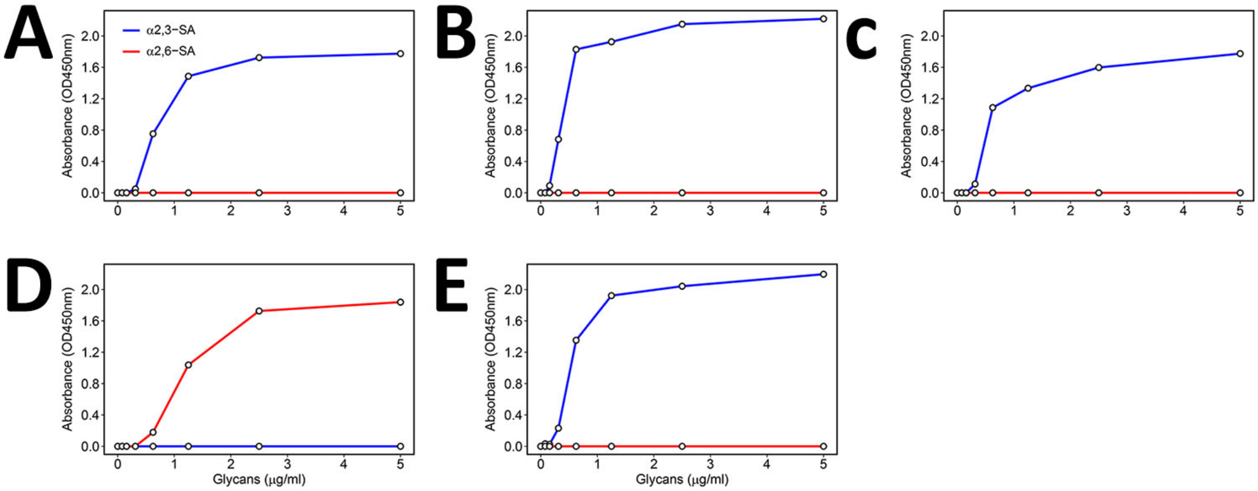

Appendix 2 Figure 2. Receptor binding properties for novel H5N1 viruses identified in this study. Receptor binding properties for (a) Ws/NC/AK1-O/2021(H5N1), (b) Ws/NC/AK2-O/2021(H5N1), (c) Bs/EC/74-Lg/2021(H5N1), (d) A/California/04/2009(H1N1), and (e) A/Vietnam/1194/2004(H5N1) virus to α-2,3-linked (blue) and α-2,6-linked (red) sialic acids (SAs), respectively. The A/California/04/2009(H1N1) and A/Vietnam/1194/2004(H5N1) viruses were used as controls with known receptor binding preference to human-type (α-2,6-linked SA) and avian-type receptor (α-2,3-linked SA), respectively. The values represented by points are the mean of three readings on the absorbance value.

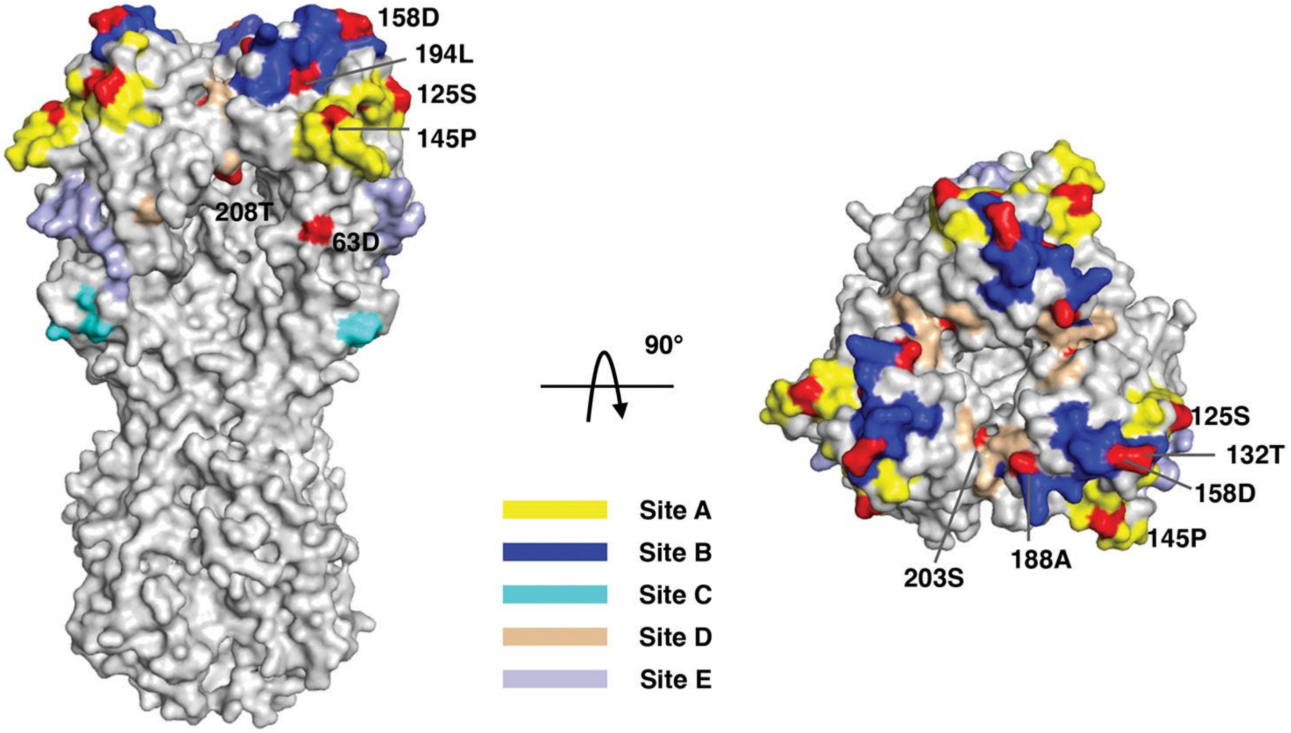

Appendix 2 Figure 3. Trimer structure of the ectodomain of HA molecule of the Ws/NC/AK1-O/2021(H5N1) estimated by homology modeling. Five major antigenic sites A–E given antigenicity study on H3 influenza were colored by different colors. The key amino acids on antigenic sites of Ws/NC/AK1-O/2021(H5N1) are colored in red. All positions are shown in H3 numbering. PDB ID of the template used in homology modeling is 7DEB.

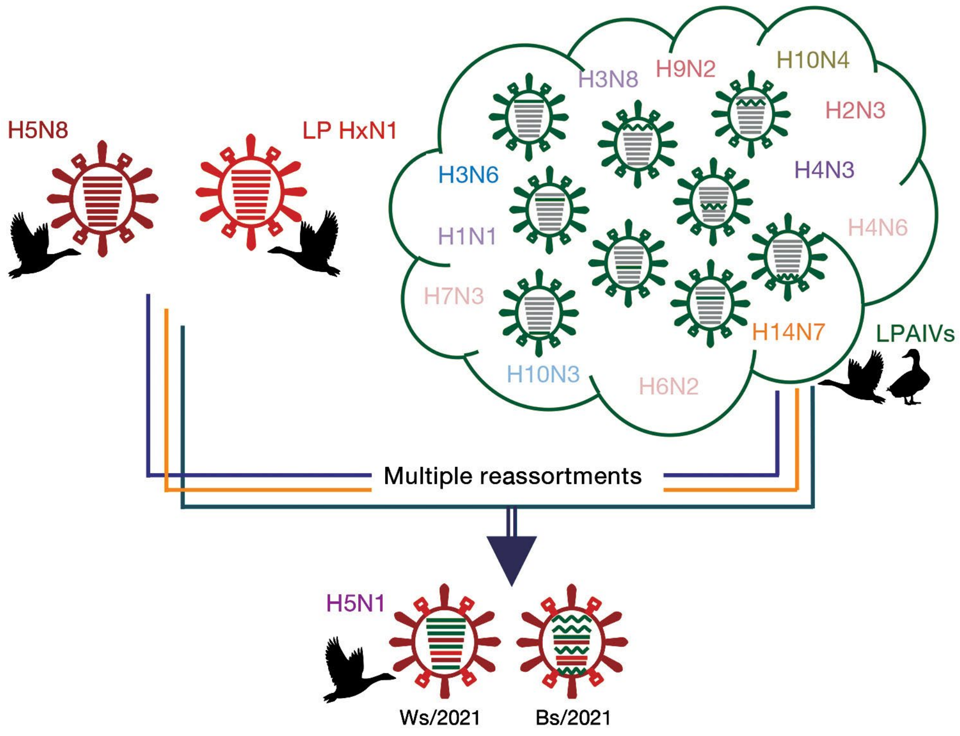

Appendix 2 Figure 4. Evolutionary pathways of two H5N1 reassortants identified in this study. The horizontal bars in the virus particle represent the eight gene segments (from top to bottom: PB2, PB1, PA, HA, NP, NA, MP, and NS). Gene segments in descendent viruses are colored according to potential parent viruses. The Ws/2021-like and Bs/2021-like H5N1 reassortants identified in this study resulted from multiple reassortments between the H5N8 HPAIVs from wild waterfowl and the LPAIVs from wild waterfowl or ducks. The reassortants obtained their HA and MP genes from the H5N8 HPAIVs, NA genes from the LPAIV HxN1 virus, and PB2, PB1, PA, NS, and NP genes from multiple LPAIV pools.

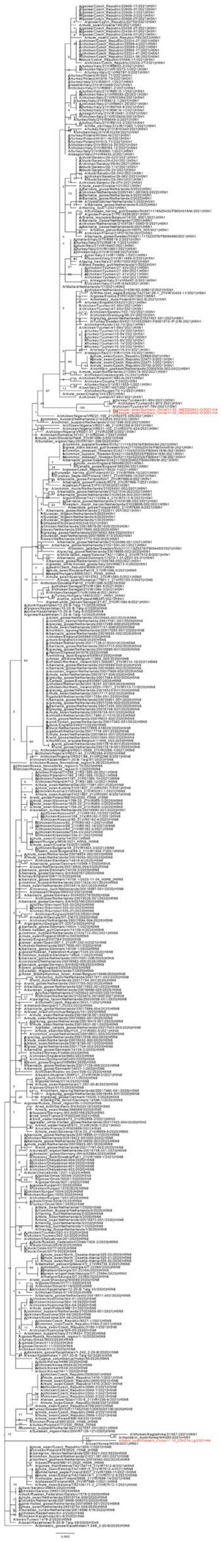

Appendix 2 Figure 5. Phylogenetic tree of HA genes of the novel H5N1 viruses and their reference viruses with the most genetic identities. The viruses identified in this study are colored in red.

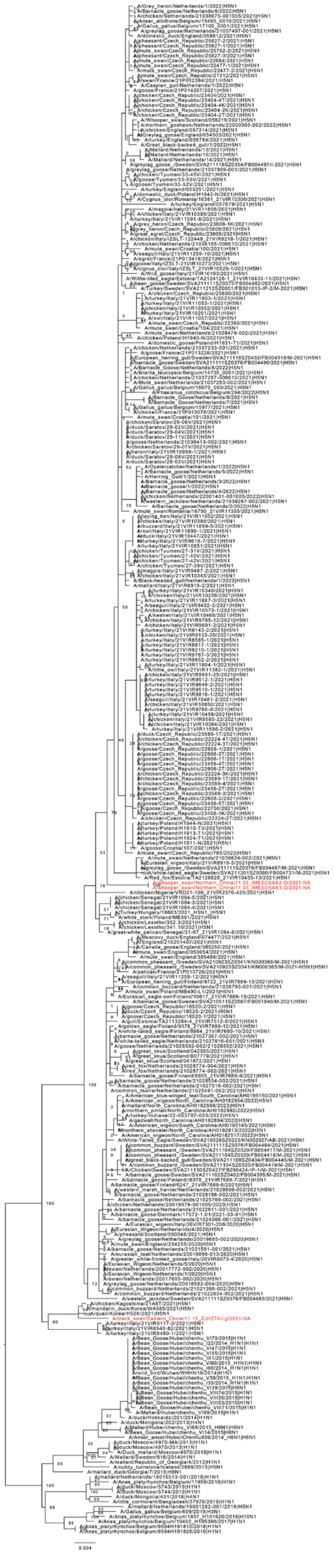

Appendix 2 Figure 6. Phylogenetic tree of NA genes of the novel H5N1 viruses and their reference viruses with the most genetic identities. The viruses identified in this study are colored in red.

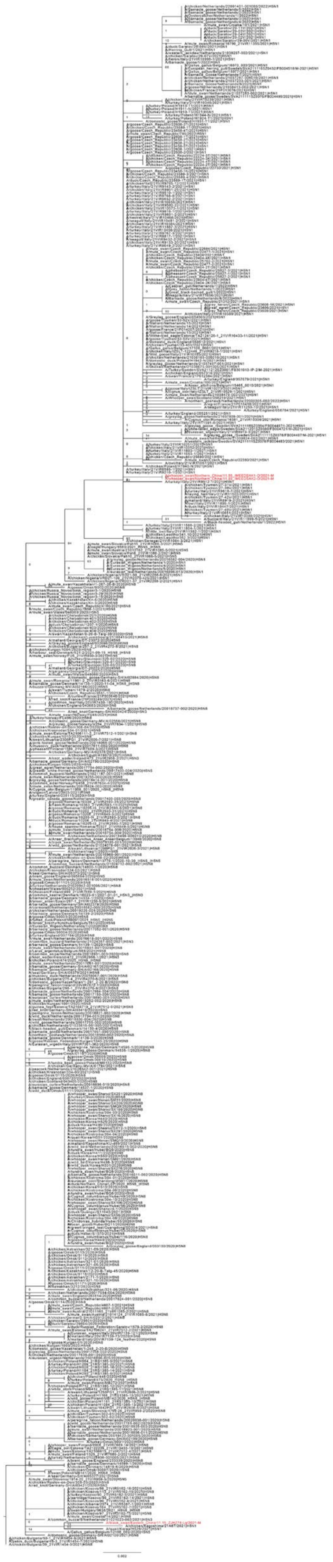

Appendix 2 Figure 7. Phylogenetic tree of MP genes of the novel H5N1 viruses and their reference viruses with the most genetic identities. The viruses identified in this study are colored in red.

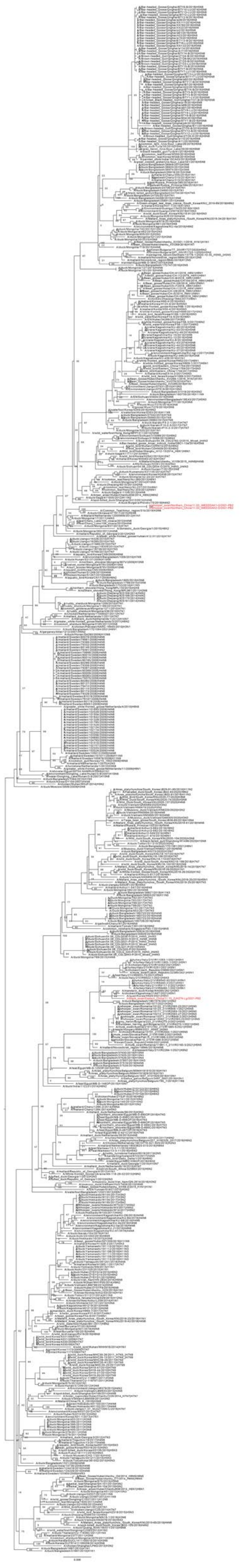

Appendix 2 Figure 8. Phylogenetic tree of PB2 genes of the novel H5N1 viruses and their reference viruses with the most genetic identities. The viruses identified in this study are colored in red.

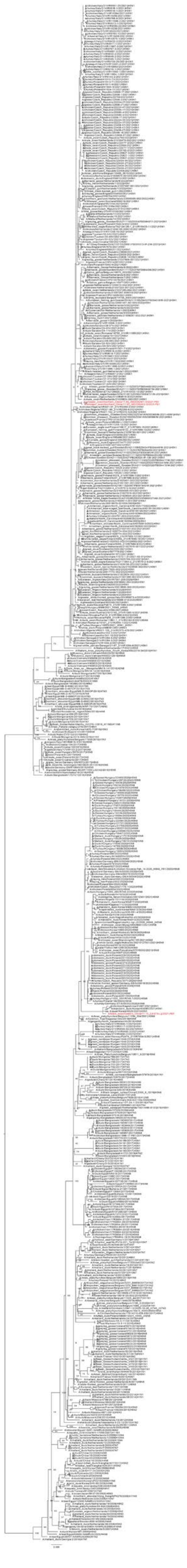

Appendix 2 Figure 9. Phylogenetic tree of PB1 genes of the novel H5N1 viruses and their reference viruses with the most genetic identities. The viruses identified in this study are colored in red.

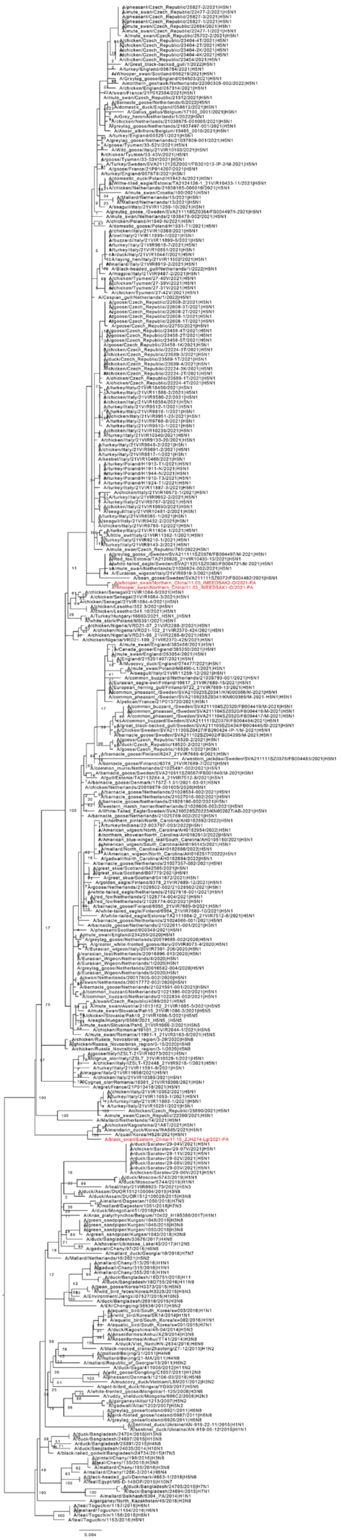

Appendix 2 Figure 10. Phylogenetic tree of PA genes of the novel H5N1 viruses and their reference viruses with the most genetic identities. The viruses identified in this study are colored in red.

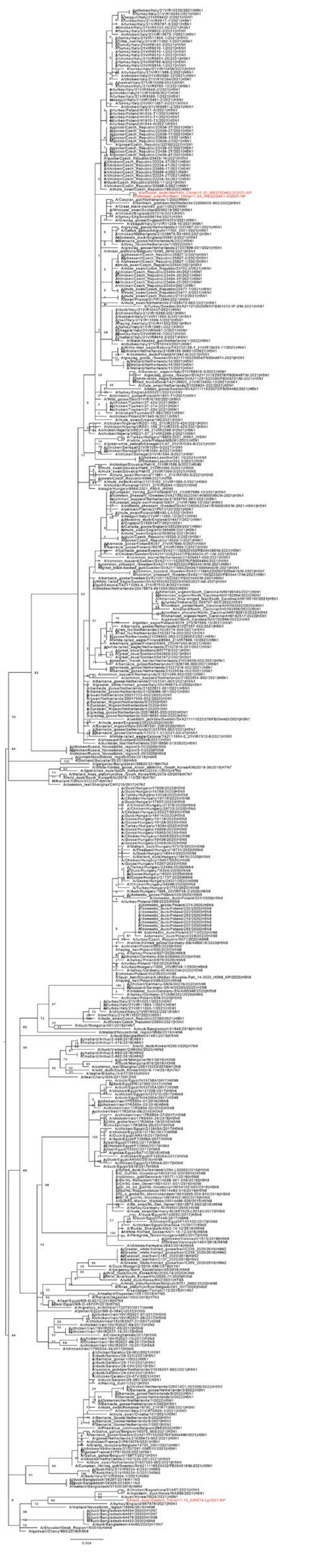

Appendix 2 Figure 11. Phylogenetic tree of NP genes of the novel H5N1 viruses and their reference viruses with the most genetic identities. The viruses identified in this study are colored in red.

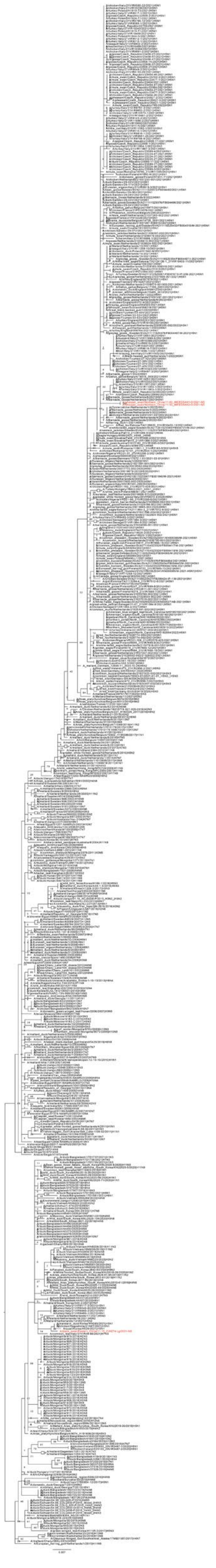

Appendix 2 Figure 12. Phylogenetic tree of NS genes of the novel H5N1 viruses and their reference viruses with the most genetic identities. The viruses identified in this study are colored in red.

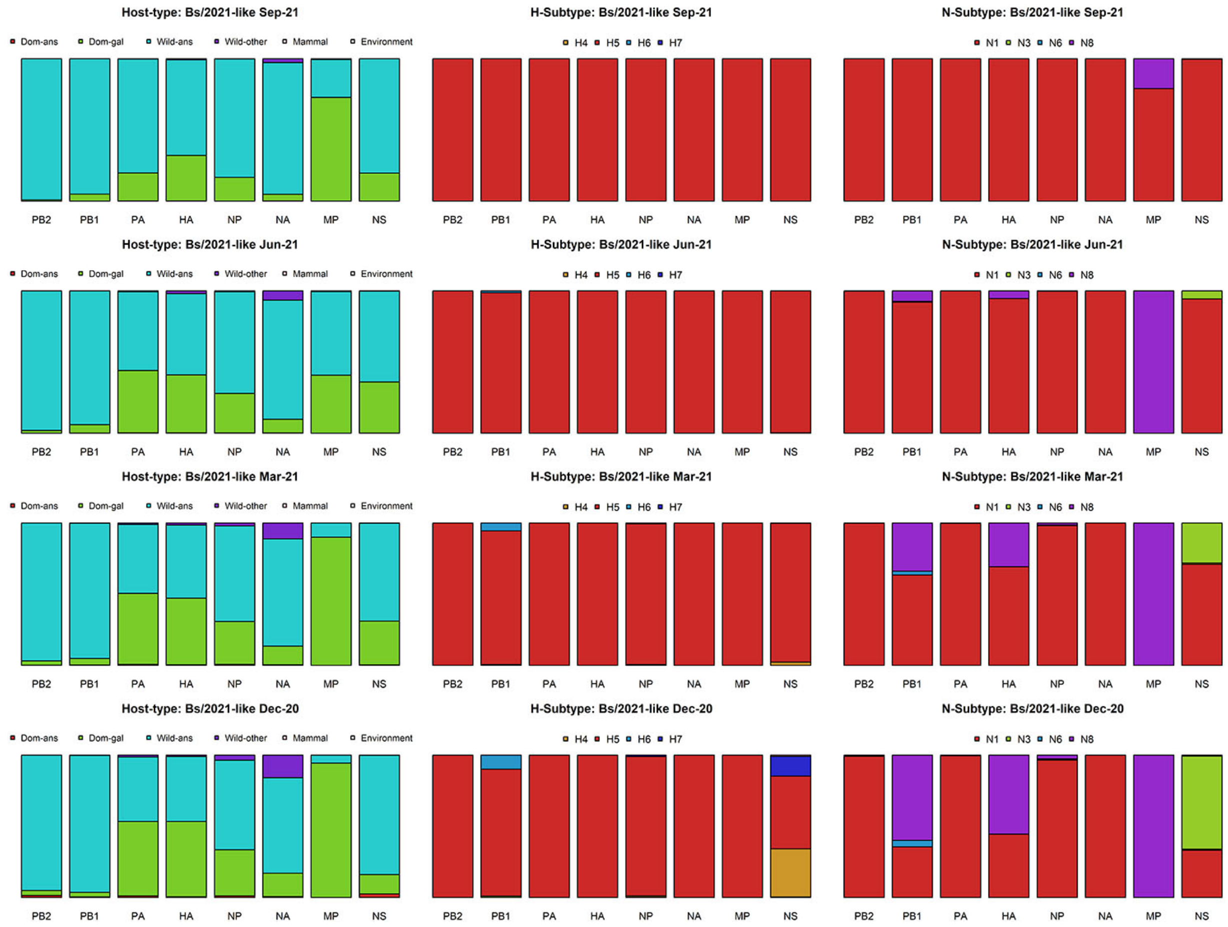

Appendix 2 Figure 13. Inferred ancestral hosts, HA and NA types of Bs/2021-like H5N1 reassortant. The ancestral host and HA/NA type estimations were adjusted by interpolating by every 3 months between December 2020 and November 2021. For each segment, stack bars represent the posterior probabilities of each type of the discrete traits (see legend).

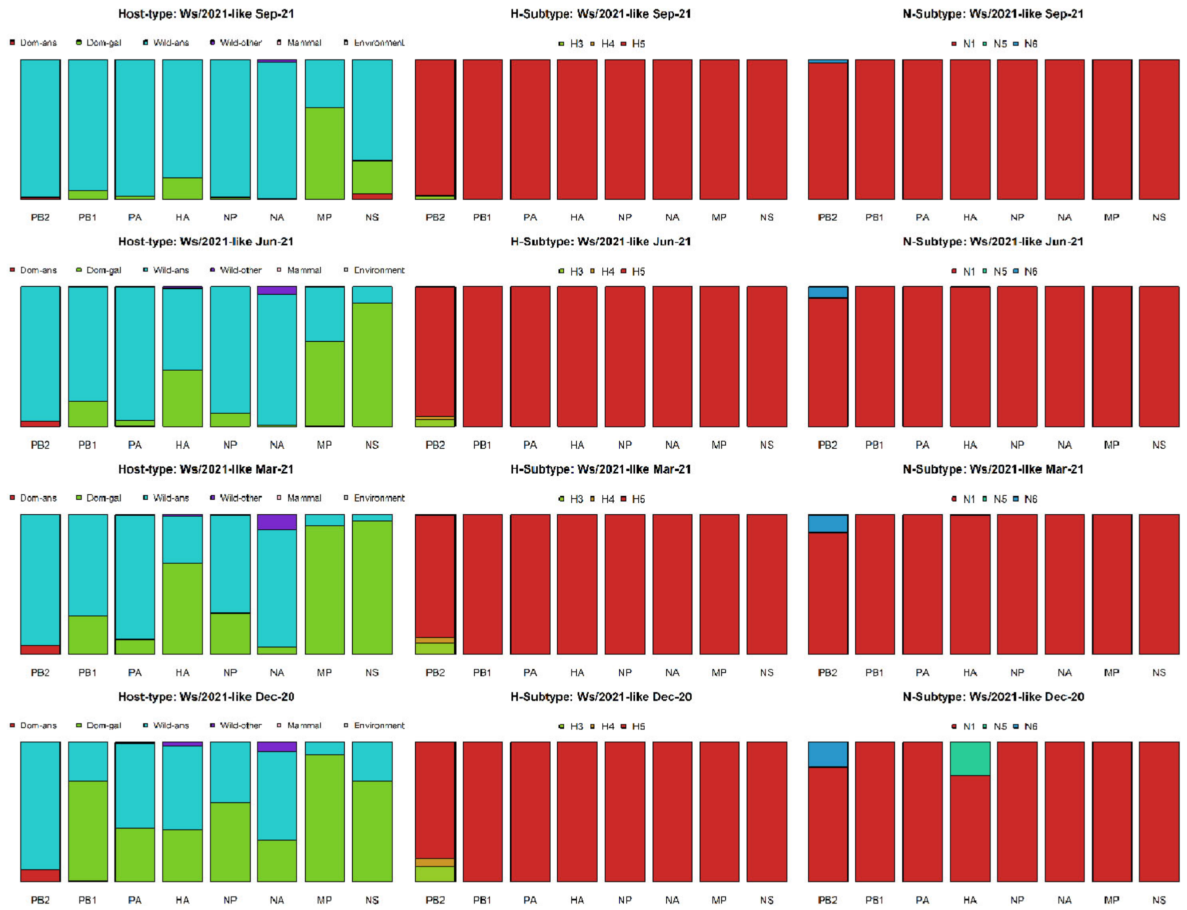

Appendix 2 Figure 14. Inferred ancestral hosts, HA and NA types of Ws/2021-like H5N1 reassortant. The ancestral host and HA/NA type estimations were adjusted by interpolating by every 3 months between December 2020 and November 2021. For each segment, stack bars represent the posterior probabilities of each type of the discrete traits (see legend).
